# Evolution of dynamic, biochemical, and morphological parameters in hypothermic machine perfusion of human livers: A proof-of-concept study

**DOI:** 10.1371/journal.pone.0203803

**Published:** 2018-09-14

**Authors:** H. Abudhaise, B. R. Davidson, P. DeMuylder, T. V. Luong, B. Fuller

**Affiliations:** 1 UCL Division of Surgery and Interventional Sciences, Royal Free Hospital, London, United Kingdom; 2 Organ Recovery Systems, Zaventem, Belgium; 3 Department of Cellular Pathology, Royal Free London NHS Foundation Trust, London, United Kingdom; Indiana University, UNITED STATES

## Abstract

**Introduction:**

Hypothermic machine perfusion (HMP) is increasingly investigated as a means to assess liver quality, but data on viability markers is inconsistent and the effects of different perfusion routes and oxygenation on perfusion biomarkers are unclear.

**Methods:**

This is a single-centre, randomised, multi-arm, parallel study using discarded human livers for evaluation of HMP using arterial, oxygen-supplemented venous and non-oxygen-supplemented venous perfusion. The study included 2 stages: in the first stage, 25 livers were randomised into static cold storage (n = 7), hepatic artery HMP (n = 10), and non-oxygen-supplemented portal vein HMP (n = 8). In the second stage, 20 livers were randomised into oxygen-supplemented and non-oxygen-supplemented portal vein HMP (n = 11 and 9, respectively). Changes in dynamic, biochemical, and morphologic parameters during 4-hour preservation were compared between perfusion groups, and between potentially transplantable and non-transplantable livers.

**Results:**

During arterial perfusion, resistance was higher and flow was lower than venous perfusion (p = 0.001 and 0.01, respectively); this was associated with higher perfusate markers during arterial perfusion (p>0.05). Supplementary oxygen did not cause a significant alteration in the studied parameters. Morphology was similar between static and dynamic preservation groups. Perfusate markers were 2 fold higher in non-transplantable livers (p>0.05).

**Conclusions:**

Arterial only perfusion might not be adequate for graft perfusion. Hepatocellular injury markers are accessible and easy to perform and could offer insight into graft quality, but large randomised trials are needed to identify reliable quality assessment biomarkers.

## Introduction

Hypothermic machine perfusion (HMP) has gained interest as a potential means to improve the outcome of marginal livers and allow viability assessment in high-risk grafts [1–5]. Despite this potential of HMP, data on viability biomarkers is inconsistent and none of the investigated biomarkers is able to differentiate between viable and non-viable livers [1, 6, 7].

Although HMP allows for assessment of dynamic perfusion parameters, there is no conclusive evidence that dynamic parameters correlate with graft quality, and the progression of these parameters differs significantly between studies; while recent clinical studies reported stable hepatic artery (HA) and portal vein (PV) pressures during HMP [2, 3], others observed a drop in HA resistance and increased flow during HMP, suggesting relaxation of the arterial walls and improved microcirculation [8–11]. Several perfusate markers have been investigated, but none were able to assess graft viability reliably, and different cut-off values were proposed to indicate the severity of reperfusion injury [3, 7, 10]. Histological examination is limited to steatosis assessment in clinically suspicious grafts [12–14], but it is not currently used for viability assessment due to its invasive nature, limited amount of tissue, and lack of a morphological standard for viability assessment [1, 7]. In addition, human studies reported controversial results on the morphology of livers preserved with HMP and static cold storage (SCS) [2, 15–17]. Finally, the benefits of supplementary oxygen during short-term end ischemic hypothermic perfusion have been documented in pre-clinical and clinical studies [4, 5, 18], but the effect of supplementary oxygen on injury markers during human liver HMP is unclear.

The aim of this study is to evaluate the progression of dynamic, biochemical and histological markers during HMP in the human liver, comparing arterial, oxygen-supplemented venous and non-oxygen-supplemented venous perfusion, and to study these markers in relation to organ quality.

## Materials and methods

This is single-centre, randomised, multi-arm parallel group study.

### Participants

Ethical approval for the study was obtained from the North London Regional Ethics Committee 3 (reference no. 10/H0709/70), and confirmed with the National Health Service Blood and Transplant (NHSBT) Organ Donor Transplant committee. None of the transplant donors were from a vulnerable population and all donors or next of kin provided written informed consent that was freely given.

The study involved 45 sequential livers procured for transplantation by different organ procurement teams across the UK between February 2012 and June 2014. All livers were retrieved using the standard clinical procurement protocols adopted by the UK National Organ Retrieval Service (NORS) (http://www.odt.nhs.uk/national-organ-retrieval-service/policies-and-reports), but were found to be unsuitable for transplantation by UK transplant centres due to a combination of donor graft and recipient factors (Table 1). Livers were transported from the retrieval hospital to our research centre packed in ice and stored in University of Wisconsin (UW) solution. Only whole livers from deceased donors were used; one split liver was excluded from the study.

**Table 1:** Donor graft and recipient factors associated with graft decline for transplantation.

Reason for declining the graft	Frequency	Percentage %	
Old age	3	7	*Donor related*
Alcohol abuse	2	4	*Donor related*
Other donor factors	3	7	*Donor related*
Long WIT	5	11	*Graft related*
Long CIT	1	2	*Graft related*
Steatosis (moderate-severe)	18	39	*Graft related*
Fibrosis	5	11	*Graft related*
Caeliac artery disease	2	4	*Graft related*
Poor flow	1	2	*Graft related*
Poor LFT	5	11	*Graft related*
Suspicious liver lesions	2	4	*Graft related*
Capsular tear	2	4	*Graft related*
No suitable recipient	3	7	*Recipient related*

Characteristics of the liver grafts in this study, showing the documented reasons for graft decline for transplantation. Less common donor factors (under other donor factors in the table) included a single occurrence of intra-abdominal sepsis, high BMI, and hemorrhagic pancreatitis. Poor flow during the initial on-table flush by the retrieval team was documented as a factor in one liver. DCD, donation after cardiac death; long warm ischaemia time (WIT) > 30 minutes; long cold ischaemia time (CIT) > 12 hours; LFT, liver function tests.

### Randomisation

The original prototype of the Organ Recovery Workstation^®^, based on the Lifeport Kidney Transporter machine (Organ Recovery Systems, Zaventem, Belgium), did not incorporate an oxygen delivery system, which was later integrated during the study period. Consequently, an oxygenated perfusion group could not be randomised at the start of the study, and was added during the second stage of the study. Livers were randomised at 2 stages: stage 1, 25 livers were randomised into static cold storage (SCS, n = 7), arterial perfusion through the hepatic artery (AP, n = 10), and non-oxygen-supplemented venous perfusion through the portal vein (nOVP, n = 8). Arterial perfusion was studied herein to further assess this perfusion modality following an earlier published study by our team [16], which showed equivalence of endothelial preservation using different perfusion routes. In stage 2, 20 livers were randomised into oxygen-supplemented perfusion (OVP, n = 11) and non-oxygen-supplemented venous perfusion (nOVP, n = 9). The nOVP groups in stages 1 and 2 of the study were similar in characteristics and perfusion settings. Simple randomisation was performed using a computer-generated list of random numbers and the allocation sequence was concealed in numbered, opaque, and sealed envelopes. Sample size calculation was not performed.

### Interventions

Livers in the SCS group were submerged in 2 L of KPS-1 and placed in the icebox for 4 hours. Although KPS-1 is not conventionally used for static preservation, it was used for SCS herein to reduce preservation solution variance between groups. Livers in the AP group were cannulated through the HA (Lifeport SealRing^™^ 10x35 cannula), and livers in the nOVP and OVP groups were cannulated through the PV (Lifeport Straight 8mm cannula). HMP livers were connected to the non-pulsatile roller pump of the Organ Recovery Workstation, and perfused with 2 L of recirculating KPS-1 through the HA or PV, at 30 mmHg or 7 mmHg, respectively. The preservation period of 4 hours was hypothesised as adequate to observe dynamic and biochemical changes during HMP.

For the OVP group, 100% oxygen was administered at a fixed rate of 0.5 L/min using a membrane oxygenator (Dideco Kids D100, Sorin Group Italia, Mirandola, Italy) connected to the perfusion circuit, achieving a perfusate pO_2_ of 735.1 mmHg (724.1–791.4).

Hypothermia was maintained between 4–8° C using a heat exchanger (Polystat temperature controller, Cole-Parmer UK) and a built-in iced-water container around the perfusion bowl. Similar to clinical studies by Guarrera [2, 3], no metabolic adjustment was undertaken.

### Outcome measures

Dynamic perfusion parameters (flow and vascular resistance) were continuously measured in the HA (AP group) and PV (nOVP and OVP groups) using built-in sensors in the Organ Recovery Workstation and recorded hourly. Hepatocellular injury enzymes (alanine transaminase, ALT; aspartate transaminase, AST) were measured in the perfusate using Roche P Module analyser (Roche Diagnostics Limited, UK), immediately before HMP, then hourly afterwards. For morphological assessment, a wedge biopsy was taken from segment 4 and segment 7 before and after 4-hour preservation (SCS and HMP groups). The rationale for using biopsies from 2 different segments of the liver is based on evidence published elsewhere [19]. Paraffinised, hematoxylin and eosin (H&E) stained liver biopsies were examined under light microscopy by a liver histopathologist. Assessors of biochemistry and histology samples were blinded to the allocation groups. A scoring system previously published by Vekemans et al. [17] was used to score morphological changes, based on enlargement of the space of Disse, sinusoidal dilatation, coagulation necrosis, congestion, architectural damage, and neutrophil infiltration.

### Statistical analysis

Data was analysed using IBM SPSS Statistics for Macintosh, Version 20.0. Armonk, NY: IBM Corp. Continuous data was presented as median (interquartile range) and categorical data was presented in numbers and percentages. Non-parametric tests were used due to small sample size and abnormal distribution. Kruskal-Wallis (KW) and Friedman tests were used to compare differences between and within groups, respectively. When KW test or Friedman test showed a significant difference, a pairwise comparison using Mann-Whitney test or Wilcoxon test was performed. The level of significance was set at p < 0.05.

## Results

### Donor graft characteristics

Characteristics of the donor grafts (n = 45) are presented in Table 2. There were no significant differences in donor graft variables (age, heart-beating status, cold and warm ischaemia times, and macrosteatosis) between the groups.

**Table 2:** Characteristics of the donor grafts used in the 2 stages of the study.

	Stage 1				Stage 2		
	SCS	AP	nOVP	Intergroup difference	nOVP	OVP	Intergroup difference
	(n = 7)	(n = 10)	(n = 8)	(p value)	(n = 9)	(n = 11)	(p value)
Age (years)	57 (50–70)	56 (51–65)	57 (49–61)	0.8	62 (48–65)	53 (44–61)	0.3
Donor type							
(DCD, %)	4, 57%	8, 80%	6, 75%	0.4	4, 44%	5, 45%	0.9
CIT (hr)	11.5 (11.0–12.3)	10.0 (9.8–14.3)	11.5 (7.0–13.8)	0.9	10.0 (10.0–14.0)	13.0 (12.0–15.0)	0.6
Total WIT (min)	23 (7–30)	19 (16–34)	24 (20–33)	0.7	23 (12–30)	22 (14–33)	0.7
Asystole WIT (min)	13 (6–19)	12 (9–14)	12 (9–14)	0.8	11 (9–14)	13 (11–15)	0.3
Steatosis %	7 (0–20)	6 (1–8)	3 (1–5)	0.8	10 (1–36)	8 (3–28)	0.9

DCD, donation after cardiac death; total warm ischaemia time (total WIT), time between withdrawal of cardiorespiratory support until cold in-situ flush; asystole warm ischaemia time (asystole WIT), time between circulatory arrest and cold in-situ flush. Steatosis was estimated as the percentage of macrosteatosis in paraffinised, H&E-stained parenchymal biopsies examined under light microscopy by a liver histopathologist. Continuous data is presented as median (interquartile range) and categorical data is presented in numbers and percentages.

### Stage 1 of the study

During the first stage of the study, livers were randomised into 3 groups: SCS (n = 7), AP (n = 10), and nOVP (n = 8).

#### Dynamic parameters during HMP

**Fig 1:**
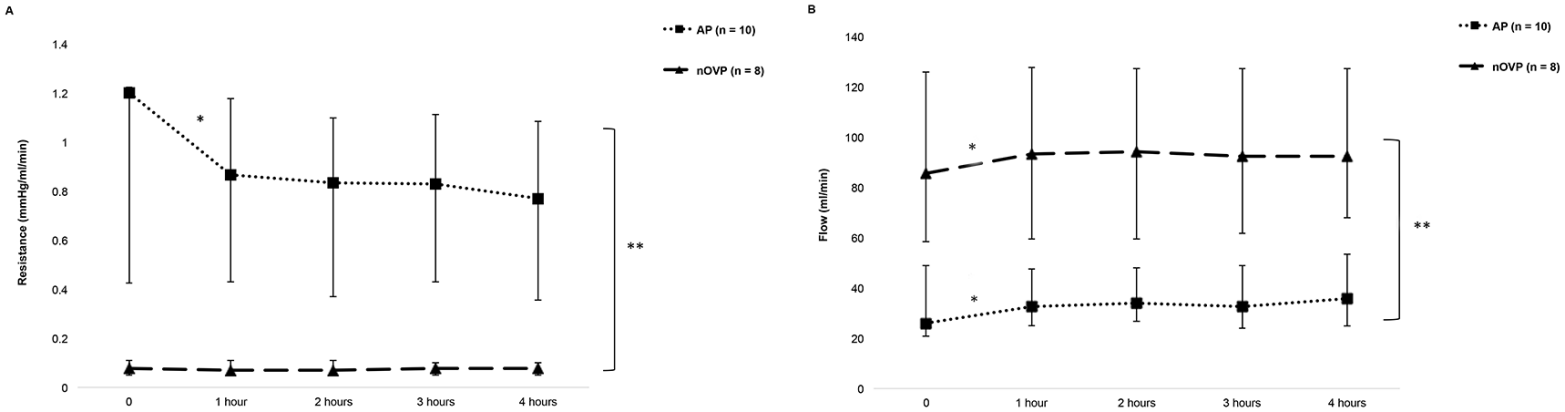
Dynamic parameters during arterial and non-oxygen-supplemented venous perfusion. (A) resistance (mmHg/ml/min) and (B) perfusate flow (ml/min) hypothermic machine perfusion. Data is shown as medians and interquartile range, the latter represented by error bars. *p < 0.05 between time points, **p < 0.05 between groups.

Resistance was stable in the nOVP group throughout HMP but decreased significantly in AP group from 0 to 1 hour (1.20 mmHg/ml/min (0.43–1.22) to 0.87 mmHg/ml/min (0.43–1.18), p = 0.01). Flow increased significantly in both groups from 0 to 1 hour (AP 26 ml/min (21–49) to 33 ml/min (25–48), p = 0.03; nOVP 86 ml/min (58–126) to 94 (59–128), p = 0.04). Resistance in the HA was significantly higher than PV (0.83 mmHg/ml/min (0.41–1.11) versus 0.07 mmHg/ml/min (0.05–0.10), p = 0.001), and perfusate flow was significantly lower in the HA compared to the PV (33 ml/min (24–48) versus 92 ml/min (59–127), p = 0.01), Fig 1.

#### Hepatocellular injury markers during HMP

**Fig 2:**
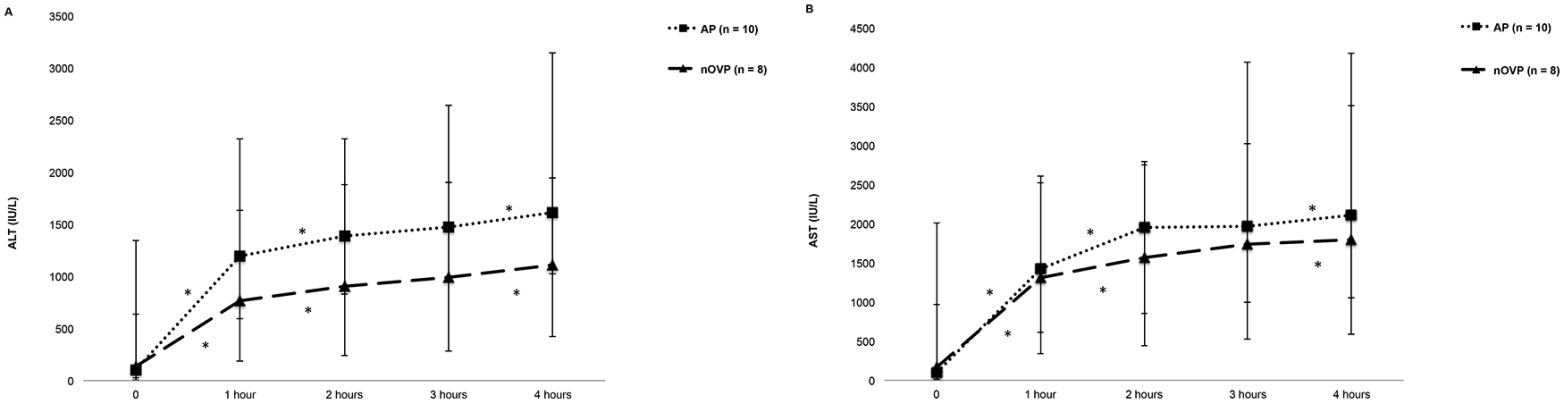
Perfusate injury markers during arterial and non-oxygen-supplemented venous perfusion. (A) perfusate ALT (IU/L) and (B) AST (IU/L) during hypothermic machine perfusion. Data is shown as medians and interquartile range, the latter represented by error bars. Enzymes increased during perfusion but there was no significant difference between groups. *p < 0.05 between time points.

ALT increased significantly throughout perfusion in the AP and nOVP groups (p ≤ 0.001 both groups) and AST levels showed a similar trend (p = 0.004 and < 0.001 for AP and nOVP groups, respectively). Of note, there was a steep rise in enzyme levels between 0 and 1 hour HMP in both groups. The enzymes were higher in AP group compared to nOVP group, but the difference was not significant (ALT, p = 0.53; AST, p = 0.50), Fig 2.

#### Morphology score in SCS and HMP

**Fig 3:**
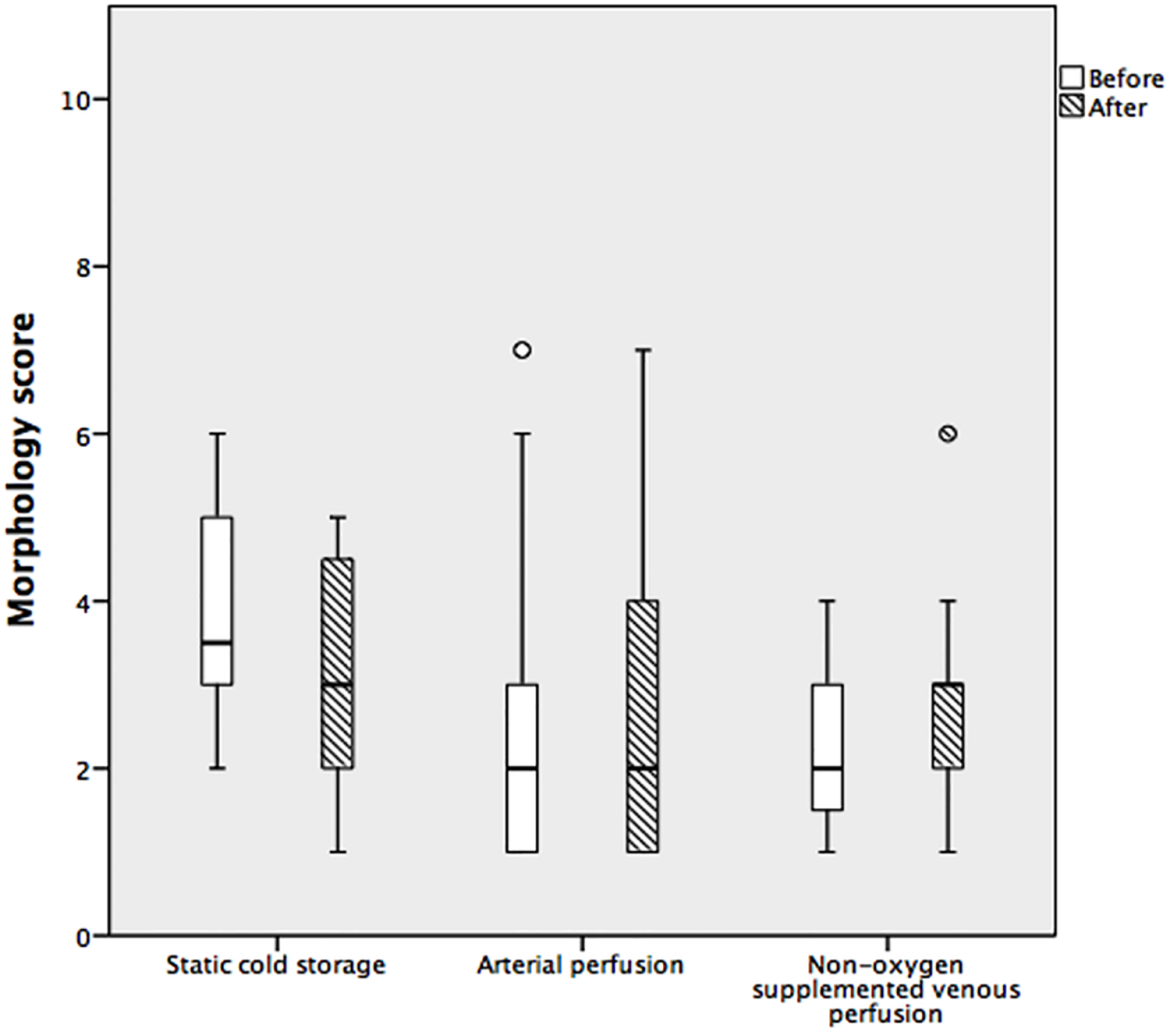
Box whisker plot of parenchymal morphology after preservation. This plot shows the distribution of morphology score values round the median, plotted before and after hypothermic machine perfusion and static cold storage. There was no significant difference in the morphology score after static cold storage, arterial perfusion, and non-oxygen-supplemented venous perfusion groups (p = 0.38). Top and bottom whiskers represent the maximum and minimum values, respectively, and circles represent outliers.

The change in morphology score was not different between groups at the end of static and dynamic preservation (p = 0.38), Fig 3.

### Stage 2 of the study

During the second stage of the study, 2 liver groups were studied, nOVP (n = 9), and OVP (n = 11).

#### Dynamic parameters during HMP

**Fig 4:**
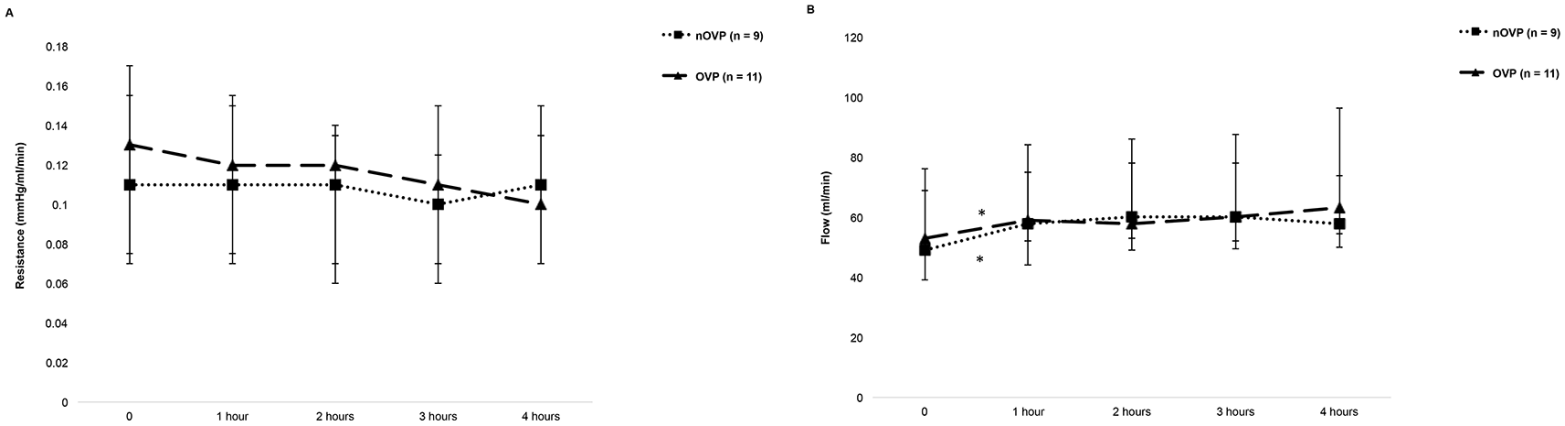
Dynamic parameters during oxygen-supplemented and non-oxygen-supplemented venous perfusion. (A) resistance (mmHg/ml/min) and (B) perfusate flow (ml/min) during hypothermic machine perfusion. Data is shown as medians and interquartile range. *p < 0.05 between time points.

Resistance was stable throughout perfusion in both groups, but flow increased significantly from 0 to 1 hour (nOVP 49 ml/min (39–76) to 58 ml/min (44–84), p = 0.01; OVP 53 ml/min (48–69) to 59 ml/min (52–75), p = 0.03). There was no significant difference in resistance and flow (p = 0.96 and 0.99, respectively) between the two groups, Fig 4.

#### Hepatocellular injury markers during HMP

**Fig 5:**
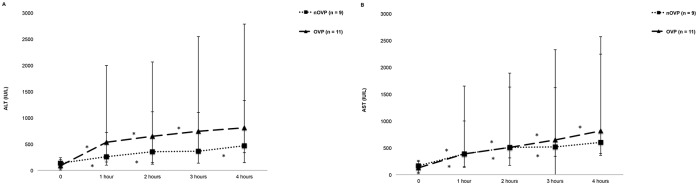
Perfusate injury markers during oxygen-supplemented and non-oxygen-supplemented venous perfusions. (A) perfusate ALT (IU/L) and (B) AST (IU/L) during hypothermic machine perfusion. Enzymes increased during perfusion but there was no significant difference between groups. Data is shown as medians and interquartile range. *p < 0.05 between time points.

ALT and AST increased significantly throughout perfusion (p < 0.001 for both enzymes in OVP and nOVP groups). There was no significant difference in ALT or AST between groups (p = 0.38 and 0.62, respectively). Fig 5.

#### Morphology score in HMP

**Fig 6:**
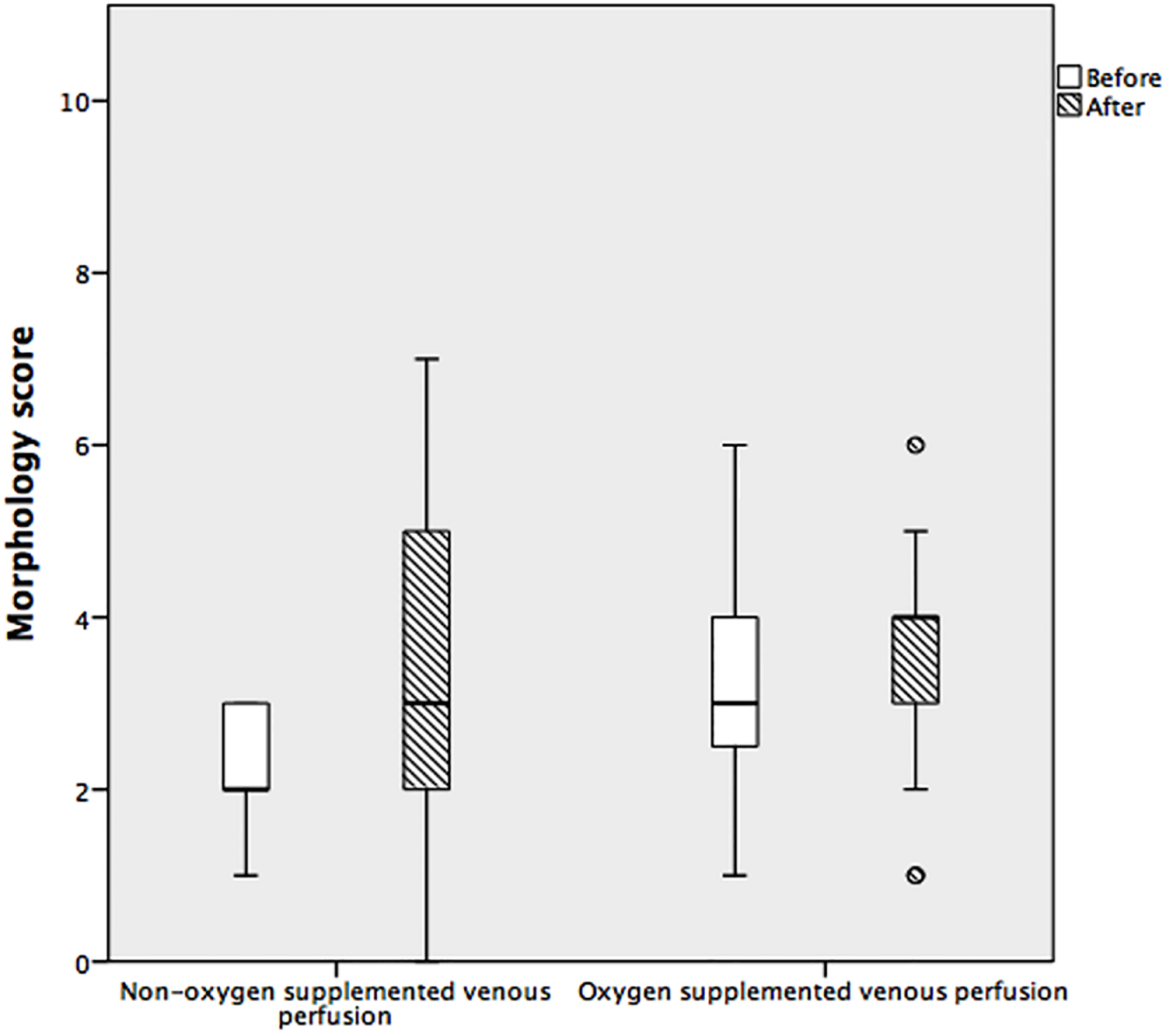
Box whisker plot of parenchymal morphology in oxygen-supplemented and non-oxygen-supplemented venous perfusion. This plot shows the distribution of morphology score values round the median, plotted before and after hypothermic machine perfusion. There was no significant difference in the morphology score between the 2 groups (p = 0.10). Top and bottom whiskers represent the maximum and minimum values, respectively, and circles represent outliers.

There was no significant difference in morphology score after OVP or nOVP, p = 0.10, Fig 6. There was no significant difference in the morphology score between segment 4 and 7 of the same liver when all samples were compared (p = 0.63).

### Comparison between potentially transplantable and non-transplantable livers

Out of 45 livers, 7 livers were potentially transplantable. 3 livers were not transplanted due to lack of suitable recipients, while 4 more livers were rejected due to suspicious liver lesions and capsular tears (2 each), (Table 1).

Potentially transplantable livers had lower median ALT and AST levels when compared to non-transplantable livers, although the difference was not statistically significant (median ALT: 348 IU/L (221–1590) vs. 653 IU/L (291–1875), p = 0.39; median AST: 512 IU/L (305–1896) vs. 1019 IU/L (429–2405), p = 0.31).

The median PV resistance in potentially transplantable livers was 0.11 mmHg/ml/min (0.08–0.17) and the median flow was 60 ml/min (49–80), compared to 0.10 mmHg/ml/min (0.06–0.14) and 67 ml/min (52–94) in non-transplantable livers (p = 0.25 and 0.65, respectively). None of the livers in the HA-only perfusion group were potentially transplantable, so a comparison could not be performed. Histology scores in potentially transplantable livers were not significantly different from non- transplantable livers (p = 0.97).

## Discussion

Despite the recent expansion in utilisation of HMP to improve the outcome of marginal livers in clinical practice, data on viability markers is inconsistent and no markers are yet validated. HMP is mostly performed using dual-vessel or PV-only perfusion, and feasibility of both approaches has been reported in recent clinical trials [2–4, 11] although the optimal perfusion route is not currently known. Arterial-only perfusion is less commonly used, based on evidence from animal studies showing increased graft injury and heterogeneous perfusion using the HA only [20, 21].

We found the HA and PV resistance to be similar to those documented in a previous study of discarded human livers preserved with dual-vessel perfusion at similar pressures to our experiments [10], which suggests that similar PV flows can be achieved with PV-only perfusion and questions the need for additional HA cannulation and perfusion. In addition, a recent study in rat, pig and discarded human DCD livers showed complete perfusion of the parenchyma and biliary system using hypothermic oxygenated perfusion (HOPE) using the PV only [22].

Perfusate flow increased in the first hour of HMP in all perfusion groups and there was a significant reduction of arterial resistance during the same time period. These changes in perfusion dynamics are likely to be due to “opening up” of the microvasculature and relaxation of arterial walls during HMP, similar to what has been reported previously in porcine [8, 9] and discarded human livers [10]. In line with other studies [9, 10, 23], we found the resistance to be higher and the flow lower when the graft was perfused via the hepatic artery in comparison to the portal vein. This is a reflection of the hepatic circulation in vivo where hepatic arterial circulation is characterised by higher resistance and lower flow than the portal circulation due to larger smooth muscle content in the tunica media layer in the arterial walls.

The early dynamic changes in our study were associated with a steep rise of hepatocellular injury markers in most liver groups during the first hour of HMP, suggesting an accelerated release of hepatocellular enzymes into the perfusate due to flushing out of enzymes accumulated in the vascular spaces, similar to observations from a previous porcine liver study [23]. Perfusate injury markers did not stabilise after the first 1–2 hours of perfusion, but increased gradually, possibly due to pre-perfusion ischaemic injury. It is important to keep the different rates of enzyme release in mind when collecting perfusate samples, as initial samples might not reflect the severity of graft injury. In addition, hepatocellular injury markers were higher in single-vessel arterial perfusion compared to single-vessel venous perfusion, although the difference was not statistically significant. These findings suggest that graft perfusion might not be adequate if perfusion is performed using the hepatic artery alone.

There was no difference in morphology between biopsies taken from 2 distant liver segments within each liver, and the architecture was preserved after 4-hour single-vessel perfusion, which agrees with findings from previous studies in discarded human livers [16, 17]. We did not observe significant sinusoidal dilatation or hepatocellular oedema in contrast to other researchers [8, 10, 24]. It is possible that the architectural changes in the latter studies resulted from prolonged storage (24 hours) or different machine perfusion setup [8, 10]. Based on our findings herein, and from results published previously by our team [16], it seems likely that architectural damage assessment during HMP offers limited information on the quality of grafts, although histological examination remains of importance in evaluating steatosis severity, degree of fibrosis, and for the assessment of suspicious lesions found during organ retrieval.

When potentially transplantable livers were compared to non-tranplantable livers, hepatocelullar markers in the perfusate were 2 fold higher in the latter group, but the difference was not statsticaly signficant. In another proof-of-concept study, Monbaliu [10] reported that perfusae AST distinguished between potentially transplantable and non-transplantable livers, and noted that AST and LDH levels were signficantly higher in non-transplantable livers. Although our results match the enzyme levels documented for potentially transplantable livers in the latter study, they do not show the same level of significant difference, most likely due to the much higher AST levels observed in non-transplantable livers in Monbaliu study (6–8 fold higher compared to transplantable livers). AST and ALT measured during perfusion were also found to correlate with peak post-transplant levels in the recipient in clinical trials by Guarrera [2, 3]. Consequently, it seems that perfusate hepatocellular injury markers have the potential to be used for graft quality assessment in the human liver.

Supplementary oxygen did not cause a significant alteration in dynamic, biochemical, or morphological parameters in this cohort, but the need for active oxygenation might only manifest following warm reperfusion; consequently, the effect of oxygenation on metabolism and mitochondrial function are better assessed using a normothermic reperfusion model.

We report one of the largest studies of discarded human livers, but we acknowledge that the overall sample size is relatively small due to the limited number of whole human livers available for research, which restricted additional groups from being assessed in this study, such as dual-vessel perfusion and oxygenated arterial perfusion groups. Due to technical factors related to oxygen delivery mechanism, the oxygen-supplemented perfusion group could not be randomised at the beginning of the study, but was later added in a second randomisation process. This resulted in the study being divided into 2 stages, which reduced the size of intervention groups. The study is limited by lack of clinical outcome measures, as the livers were not transplantable; the aim of the study was to describe baseline differences in biomarkers using different perfusion modalities as a proof-of-concept. An additional period of normothermic reperfusion would have been useful here, but is not a replacement for transplantation and in-situ reperfusion for optimal assessment of organ viability.

This study shows that perfusate injury markers have the potential for assessing graft quality and that perfusate markers are released at a faster rate during the initial period of perfusion, which is important if these markers are to be used for graft assessment. It is likely that single-vessel perfusion via the PV is more beneficial than HA perfusion during HMP on the basis of perfusion characteristics and perfusate injury markers. However, additional issues of importance would be the risk of vessel injury with cannulation, the effect on hepatocyte metabolism and bioenergetics including mitochondrial function and the unique importance of maintaining the peri-biliary vascular plexus that is associated with bile duct injury following transplantation. In order to evaluate the optimal perfusion settings and to identify objective markers of graft quality during perfusion, large prospective and randomised clinical trials are needed, as the current knowledge is mainly derived from small size feasibility studies. In addition, biomarker standardization and guidelines on outcome definitions are required to enable an unbiased comparison between different studies.
